# Swiss Primary Teachers’ Professional Well-Being During School Closure Due to the COVID-19 Pandemic

**DOI:** 10.3389/fpsyg.2021.687512

**Published:** 2021-07-12

**Authors:** Tina Hascher, Susan Beltman, Caroline Mansfield

**Affiliations:** ^1^Department of Research in School and Instruction, Institute of Educational Science, University of Bern, Bern, Switzerland; ^2^School of Education, Curtin University, Perth, WA, Australia; ^3^School of Education, The University of Notre Dame Australia, Fremantle, WA, Australia

**Keywords:** well-being, teacher, school, resilience, school closure, COVID-19

## Abstract

During sudden school closures in spring 2020 due to the COVID-19 pandemic, teachers had to move to distance teaching. This unprecedented situation could be expected to influence teacher well-being and schools as organizations. This article reports a qualitative study that aims at understanding how changes in teachers’ professional lives that were related to school closure affected Swiss primary teachers’ professional well-being. In semi-structured online-interviews, 21 teachers from 15 schools sampled by snowball method reported their experiences during school closure and distance teaching and how this situation influenced their professional well-being. Results showed that medium to high levels of teacher well-being could accompany a general negative evaluation of the move to distance teaching. Factors such as high work-load, social distancing and feelings of lack of competence and self-efficacy were among the most aversive aspects of distance teaching and associated with deteriorating professional well-being. Among a plethora of factors that supported teachers in maintaining their well-being, contextual work-related aspects such as school resources, collegial support or leadership support along with individual aspects such as resilience, coping strategies, and clear work structures were important. Additionally, it was found that teacher well-being was nourished by positive experiences with the new forms of distance teaching and feelings of professional mastery. Despite methodological limitations (snowball sampling, retrospective interviews), the findings of this study could inform schools and authorities about what is needed to support teacher well-being and might help to develop organizational strategies that aim at preventing harmful declines in teacher well-being during challenging and difficult times such as a pandemic.

## Introduction

The main role of schools is the formal education of children and adolescents. Schools should support students in acquiring academic knowledge and skills, flourishing and striving as lifelong learners, as well as becoming responsible members of a sustainable and fair future in globalized societies. These challenging and demanding tasks call for optimal functioning and a highly committed and effective teaching force in schools. Teacher well-being is a crucial resource for the academic success of students and for school effectiveness as it correlates positively with feelings of competence and relatedness with students (Collie et al., 2016), can affect teaching practices and student learning (Turner and Thielking, 2019), and temper effects of stress (Hung et al., 2016). However, international studies show that teacher well-being is at risk and that schools around the world experience teacher shortage and attrition (e.g., The Badass Teachers Association (BATs), 2017; Education Support, 2019; OECD, 2020). Teachers experiencing diminished well-being at school are less able to provide high quality teaching and tend to leave the profession earlier (OECD, 2021). Teacher attrition due to low levels of well-being, in turn, may undermine school quality. Concurrently, a teaching profession whose demands are known to challenge individual work-life balance fails to attract people to work in schools.

The outbreak of the COVID-19 pandemic in the beginning of 2020 dramatically changed lives in schools. After schools were fully or partly closed, schools as organizations as well as every individual principal, teacher, and school faculty member had to adapt to a predominantly new and potentially threatening situation. Never before, has any societal change had such a strong influence on schools. Never before, has any societal change impacted all members of a school community and in nearly every country. In Switzerland, where the vast majority of structures, organizations and processes of the education system are based on face-to-face education, school closure led to a very unfamiliar situation, specifically for teachers and learners in compulsory education. No teacher has been trained for the move to online teaching; no student has been prepared for learning from home; no parent has been prepared for home-schooling; no school has been equipped for the technical and pedagogical support of distance learning.

It is likely that this situation has influenced teacher well-being. Along with daily demands, teachers had to face new challenges with distance teaching, social distancing, technically driven communication with children and parents as well as with school colleagues and principals. Aside from worries about their own health, teachers were expected to provide the best education to children. Along with regular high expectations of the teaching force, teachers had to manage teaching and learning under even harder conditions due to school closure during the pandemic (OECD, 2021). Given the high demands on teachers, our study aims to understand how school closure due to the COVID-19 pandemic influenced primary teacher well-being and what could be learned from the results for the support of teacher well-being in primary schools. In order to get a deeper insight into teachers’ perspectives, we wanted to emphasize their individual experiences during school closure and distance teaching in primary education. Both demands and potential resources during school closure were addressed and investigated regarding their impact on primary teacher well-being.

### Teacher Well-Being

Teacher well-being has been acknowledged to be crucial for teachers’ lives as low levels of teacher well-being are a threat to teacher health (e.g., Gray et al., 2017; School Mental Health Group, 2019). It is a major driver of teaching quality (Duckworth et al., 2009) and student achievement (Branand and Nakamura, 2017). Given the high workload and professional responsibilities that teachers face, their well-being is a precious resource for high quality teaching and supports teachers’ professional ability. Also, for schools as organizations, teacher well-being is of utmost importance. In helping to prevent emotional exhaustion and burn-out, teacher well-being may contribute to prevent an individual’s intentions to leave the profession and to a reduction in teacher attrition (e.g., Renshaw et al., 2015; McCallum et al., 2017; Skaalvik and Skaalvik, 2018). Understood as a symbol of predominantly positive emotions and cognitions toward the profession and individual work, teacher well-being supports teachers’ optimal functioning in and commitment to school (Creemers and Reezigt, 1996). Teacher well-being is important for school improvement processes and successful educational governance (Sutton and Wheatley, 2003; Zembylas, 2010) whereas low levels of teacher well-being can hamper school improvement and educational reforms and lead to higher rates of teacher absenteeism (Parker et al., 2012; Education Support, 2019; Turner and Thielking, 2019). In sum, teacher well-being has been valued as a precondition as well as indicator of a successful fulfillment of the professional role and the meaningfulness of professional work as a teacher (e.g., Deci and Ryan, 2008). As teacher well-being is directly and indirectly linked to school effectiveness, it bears high relevance for schools as organizations that teach and educate future generations. However, among a plethora of studies that investigate teacher well-being studies seem to forgo the differentiation between different school type or to tailor research questions to the specific demands of primary education. Thus, less is known about teacher well-being in primary education that shows specific characteristics such as teaching children in an age range from 5 to 12 years, heterogeneous classroom composition, inclusive education, teaching goals that aim at basic education, and generalist teacher education.

Whilst there is agreement on the importance of teacher well-being, differences exist regarding the question of how teacher well-being is conceptualized. Definitions and operationalizations range from positive aspects of psychological functioning such as job satisfaction, intrinsic motivation, and positive emotions at work to worries and complaints related to work such as negative emotions, exhaustion, or stress. Also, various forms of well-being can be differentiated such as a psychological, physical, or social well-being (e.g., WHO, 1946), hedonic and eudemonic well-being (Ryan and Deci, 2001) or workplace well-being that is defined as consisting of organizational well-being and subjective well-being (Burns and Machin, 2013). This heterogeneity is based on the conceptualization of well-being as a multidimensional construct that has found its early roots in Diener (1984) definition of subjective well-being as a composite of satisfaction as well as positive and negative affect. Over the years, well-being has reached increasing multi- and transdisciplinary attention as well-being is expected to influence various dimensions of individual and societal life (McCallum et al., 2017). This multi- and transdisciplinary view has contributed to the enhancement but also blurring of the construct of well-being.

With regards to the teaching profession, researchers from different fields of psychology (e.g., Seligman, 2002; Collie et al., 2016; Royer and Moreau, 2016), education (e.g., Laine et al., 2018), and health sciences (e.g., Sadick and Issa, 2017) aim for a greater understanding of teacher well-being. Teacher well-being can be described through a variety of indicators and factors. Frequently, positive factors (e.g., positive emotions, satisfaction, or self-efficacy beliefs) as well as negative factors (e.g., negative emotions, stress, or complaints) are recognized as defining components. Due to the co-existence of positive and negative factors, teacher well-being can be defined by their relationship and be understood as a “positive imbalance” that represents a dominance of positive over negative factors. The more pronounced this difference between the positive and negative factors, the higher the perception of well-being. Positive emotions, cognitive evaluations and worries, or complaints have been identified as core elements of teacher well-being (Hascher, 2012).

Interestingly, across a variety of studies it has been found that well-being and satisfaction can coexist with reports of stress and demands as shown for example by low negative correlations of well-being and demands (Burns and Machin, 2013), well-being and extra duties (Collie and Martin, 2017), or well-being and workload (Lavy and Eshet, 2018). This result was even more pronounced in an earlier study with Swiss teachers (Bieri, 2006). The co-existence of positive as well as negative perceptions and evaluations of the teaching profession needs further attention. One explanation could be that teachers experience high stress at work but also exhibit resources to cope with stressors. Personal resources and capacities such as resilience (Beltman et al., 2011; Brouskeli et al., 2018), work motivation (Collie and Martin, 2017), and emotion regulation skills (Lavy and Eshet, 2018) have been found to support and protect well-being. Resources at work are also of considerable importance for teacher emotions and well-being (Salanova et al., 2006; Leithwood, 2007). Accordingly, quality of school life (Cenkseven-Önder and Sari, 2009), teacher learning climate (Shoshani and Eldor, 2016), a supportive teacher environment (Ilgan et al., 2015; Renshaw et al., 2015), or more specifically, autonomy and social support at work (Ebersold et al., 2019) have been confirmed as influential for teacher well-being. Reciprocal effects could be expected as organizational resources may influence teacher well-being, which, in turn, may influence future personal and organizational development.

Among the array of job-related factors, social relationships have been repeatedly confirmed as a major source of teacher well-being (Hascher and Waber, in review). As a social profession, teaching depends on interactions with students, colleagues and parents and teachers, and so seems to be specifically vulnerable to social factors (Gu, 2014). It has been confirmed that positive teacher-student-relationships nourish teachers’ needs for social relatedness (Spilt et al., 2011; Klassen et al., 2012; Collie et al., 2016), social support by colleagues and principals are relevant for teacher well-being (Wong and Zhang, 2014) as well as supportive leadership (Berkovich, 2018). Thus, teacher well-being can be nourished by individual social interactions as well as professional social interactions in the organization. However, little is known about the consequences for teacher well-being when teachers lose their familiar social environment and social embeddedness into school such as the COVID-19 requirement to work from home.

### School Closure in Swiss Primary Schools

As soon as the Swiss government realized the detrimental impact of the COVID-19 pandemic on public health, a lockdown that also included school closure was issued. Schools were immediately closed over a weekend in March 2020. In most schools, teachers had been informed about school closure on Friday and expected to start with distance teaching the following Monday leaving the weekend for preparation of a fully unfamiliar online teaching. During the first weeks, schools, teachers, students, and parents were left with little clarity about the duration of school closure and distance teaching and learning. Schools were informed that the Swiss government aimed at re-opening of schools as soon as possible. More precisely, the Swiss government decided on school closures on Friday, March 13th 2020. Most schools were closed from Monday, March 16th. Schools had been informed by politicians about re-opening not earlier than April 04th, and finally primary schools re-opened on May, 11th 2020. However, in the first one or more weeks schools applied the method of reduced student attendance by a daily alternate grouping (Halbklassenunterricht), i.e., each day only half of the children attended school while the other students learned online.

In Switzerland, primary education covers the years from preschool to 6th grade (K-6). Primary teachers are usually trained to teach numerous subjects for K-2 or grade 3–6, and have an average workload of 24–26 lessons a week with a fulltime position. However, part time contracts are frequent. Age-grouped and multi-grade classroom (usually two age groups in one classroom) coexist, occasionally within the same school. Thus far, little is known about the specific practice of distance teaching in primary education during school closure in Switzerland in spring 2020. However, it can be assumed that this practice has been heterogeneous in terms of quantity and quality due to the variability of work conditions in schools along with the high autonomy that is assigned to teachers in the Swiss education system. Given the shortness of time that teachers were allowed in moving to distance teaching and the lack of opportunity in organizing high quality distance teaching, it can be expected that primary teachers faced high insecurity, high stress, and high pressure to succeed. Teaching quality might have been dependent on individual teacher dispositions and competencies, and the use of technically supported distance teaching could be determined by teacher technical interests, affinity, and skills. No guidance for teacher-parent collaboration during school closure was provided. Accordingly, results from the school barometer that collected data from teachers, parents and students in Switzerland, Germany, and Austria during school closure indicated qualitative differences and a huge variety in distance teaching and home schooling (e.g., Huber and Helm, 2020). Although the sample was highly biased with an overrepresentation of German adolescent students, the collected data showed that school closure has been highly demanding for students, parents, and teachers as well as schools as organizations. A review of studies on German, Swiss, and Austrian schools during COVID-19 related school closure shows that the variety of applied teaching practices ranged from weekly task-books that students had to edit without or only minimal contact with teachers and peers to regular online meetings with the teacher and the whole classroom (Helm et al., 2021).

### The Role of School Closure for Teacher Well-Being

Well-being can be threatened by critical situations (Filipp and Klauer, 1991; Parker et al., 2012). Serious events such as a pandemic, school closure and corresponding changes in the living and working conditions of teachers can be identified as crises or critical life events. Crises or critical life events such as unemployment are risk factors with a significant negative impact on individual and collective well-being (e.g., Latif, 2010). In view of findings that teachers need to be largely free of stress and feel comfortable at work to commit themselves to innovation and change (Sisask et al., 2014), it could be assumed that teachers’ negative appraisal of working conditions under the pandemic will impede their well-being.

However, research has shown that a crisis or critical life event may even bear positive potential and also can trigger individual and collective development. There seems to be some agreement that positive effects of a critical situation or a crisis depend on how the challenging situation is appraised and whether the individual has opportunities to respond. For example, similar to the Lazarus (1966) and Lazarus and Folkman (1984) differentiation into primary and secondary appraisal, the “Job Demands-Resources Model” by Demerouti et al. (2001; Bakker and Demerouti, 2017) suggests the differentiation into two categories of job characteristics: (1) job characteristics such as high work load or emotional demanding interactions that are demanding and energy consuming; and (2) job characteristics such as opportunities for autonomy and personal growth that serve as resources for the individual and support the achievement of professional goals and development. In this model, also two opposite processes are distinguished: (1) The strain-process is characterized by the fact that challenges primarily consume energy and the high loads weaken a person’s mental and physical resources, which can lead to health problems, to low levels of well-being and to burnout. (2) The motivation-process is described as motivating because available resources lead to the task being tackled and successfully managed. The individual is seen as reactive as well as proactive in dealing with challenging, demanding and critical situations. As regards the effects of the COVID-19 pandemic, the “Job Demands-Resources Model” suggests to identify how an individual teacher appraises the burden of the challenges along with her/his personal resources to respond to this extreme situation.

In a similar vein, Podsakoff et al. (2007) and Tadić Vujčić et al. (2017) differentiated between hindrance demands (i.e., tasks and characteristics of the work that impede task fulfillment) and challenge demands (i.e., workload, responsibility, and complex tasks that also include positive aspects). They showed that hindrance demands impede autonomous work motivation challenge while demands support autonomous work motivation that in turn influence secondary teacher well-being. Moreover, it has been found in research on resilience, that stressful situations can decrease teacher well-being whereas challenging situations may even foster well-being (Beltman et al., 2011). Based on these findings, various different effects of the COVID-19 pandemic are conceivable. As a risk factor, the consequences of the pandemic could have a substantially negative effect on well-being, for example if the break with familiar practices and the completely new form of distance teaching proves to be primarily a stressor. On the contrary, understood as an opportunity for example to create new practices in remote teaching and new professional forms of collegial cooperation, the consequences of the pandemic could also promote well-being. For example, in the United States Anderson et al. (2021) found that during COVID-19 lockdown teachers’ creativity in distance teaching was related to well-being factors such as buoyancy, positive affect, and dispositional joy.

Consequences of a critical life event can affect or stimulate well-being temporarily or in the long term. A temporary influence tends to wane after a certain time and well-being settles back to its level before the event (Diener and Larsen, 1984). Current research shows that various major life events (e.g., child birth, financial loss, separation) differentially influence well-being (Kettlewell et al., 2020), i.e., how the consequences of a crisis or critical life event are perceived and which form of influence on well-being dominates is not only related to individual appraisal and individual factors such as resilience, coping strategies and individual resources but also to the nature of the event (Kettlewell et al., 2020). Moreover, as was found by Aldrup et al. (2017) contextual factors can contribute to this process of adaptation as possibly harmful effects of negative events on well-being (e.g., student misbehavior) can be moderated by social relatedness. In the case of the COVID-19 pandemic, contextual factors include, for example, policy decisions, governance by the school management, school leadership (Collie, 2021), and social support within the teaching force.

What does relevant empirical evidence indicate about how the COVID-19 pandemic influenced teacher well-being? So far, initial results from studies around the globe point to predominantly negative but also ambivalent effects. For instance, Fauzi and Khusuma (2020) found in a survey study with 45 Indonesian elementary school teachers that although the majority of teachers appreciated the opportunities given by online teaching during the pandemic, about 80% felt dissatisfied as they experienced a sense of ineffectiveness with online teaching that might have decreased their well-being. These results were confirmed in a mixed-method study with Indonesian 67 primary teachers (Rasmitadila et al., 2020) that identified challenges in online teaching, with selecting instructional strategies as well as teacher motivation, and underlined the role of teacher support (see also Putri et al., 2020). Similarly, Alea et al. (2020) identified in a survey in the Philippines that teachers felt challenged by a lack of knowledge, skills, and experience in teaching online. Gross and Opalka (2020, p. 1) found in a representative study that United States teachers had insufficiently been supported and encouraged to manage this difficult situation, leading to the result that “Far too many districts are leaving learning to chance during the coronavirus closures.”

Research also shows that teachers’ major concerns related to student cognitive and social problems during school closure. Based on an interview study with 24 teachers from English state schools, Kim and Asbury (2020) found worries about vulnerable student to predominantly intensify teachers’ anxiety and sadness. Having left student development and learning to chance could deteriorate teacher well-being. Alves et al. (2020) asked Portuguese teachers during the pandemic to compare their current professional well-being to their well-being before the pandemic. Data from 1479 teachers indicated a decrease in emotional well-being (more stressed, more tired, more work overloaded, more anxious, more pressed and more distressed along with feeling less satisfied, less motivated, less valued) and an increase in teaching difficulties regarding distance learning, use of digital platforms, and evaluation. Similarly, satisfaction with the education system and positive future perspectives declined.

Interestingly, Allen et al. (2020) could not find a decrease of teacher well-being in England measured with the Warwick-Edinburgh Mental Well-being Scale during the first weeks of the school closure. However, a closer look at the teachers’ answers revealed positive as well as negative changes. For instance, during the school closure in April 2020, teachers reported that they had more energy to spare (7 versus 34%) and felt more relaxed (15 versus 37%) in comparison to regular school time in October 2019. At the same time, teachers reported feeling less useful in April 2020 (60 versus 44%), less optimistic about the future (39 to 30%), and less interested in new things (42 to 27%).

These results call for research that aims at a deeper understanding how teachers have responded to the new working situation in the context of the COVID-19 pandemic. What could have been positively experienced during school closure in spring 2020 to support a “positive imbalance” and maintain or restore teacher well-being? It is important to understand how teachers dealt with such challenging situations, how they mastered them, and how they would advise newcomers to deal with critical life events. Challenges might be specifically pronounced in primary education that – for example – capitalizes on personalized student-teacher relationship, includes a high heterogeneity of students regarding age and family background and is less prone to the use of digital tools. Therefore, this study aims to address the following questions: How did the situation of distance teaching during school closure due to the COVID-19 pandemic influence primary teacher well-being? What did primary teachers expect from schools to support their well-being during the school closure due to the COVID-19 pandemic? What could be learned from this situation for the promotion of primary teacher well-being in schools?

## Materials and Methods

This research follows a qualitative approach. The sampling strategy was based on the snowball principle and reached about 30 primary teachers that were personally invited by email. Twenty-one primary teachers (19 women, two men, age range 26–61) from 15 schools agreed to participate in semi-structured online interviews. Participants reflected a range of work profiles, with five in their first 5 years of teaching, 13 working part time, nine class teachers, one principal; six teachers in lower primary education and 15 teachers in upper primary education, 12 in multi-grade classrooms. Teachers were interviewed after school closure between April 2020 and October 2020. The interviews usually lasted 30 min and asked about teacher professional biography and teaching roles before and during the pandemic, resources and challenges of teacher well-being during distance teaching. The present study focuses on the results of the following questions:

1. How would you rate your well-being at work out of 10 (1 = extremely low; 10 = extremely high) at the moment? What has led you to make that judgment? How typical is that rating of how you usually feel at work?
2. Has there been a time during the COVID-19 crisis when you would have rated your sense of well-being at work at a low level? Can you tell me what was happening at that time?
3. Has there been a time during the COVID-19 crisis when you would have rated your sense of well-being at work at a high level? Can you tell me what was happening at that time?
4. What advice might you give to other people working in a similar position in relation to coping with such an adverse event as the COVID-19 pandemic?
5. Is there anything else you’d like to share about your experience of working in a school during this pandemic?

These five questions were integrated into a set of 12 questions in total. At the beginning of the interview, teachers were asked to describe their professional trajectories, the roles they had undertaken in the school in 2020, to select 1 of 10 photos to illustrate their current well-being (e.g., a photo of a tree, a beach scenario, a carousel) and to explain their lay definition of teacher well-being. Then, the definition of well-being as a positive imbalance was introduced to the teachers before asking about their well-being during the pandemic (see questions 2–5 above). Toward the end of the interview, additional questions regarding what has kept them going in their job and advice for people generally enrolled in education followed. Finally, teachers were encouraged to select another of the 10 photos that would represent their desired future well-being and explain why.

All interviews were transcribed verbatim and anonymized. Data analyses followed principles of qualitative context analyses (Kuckartz, 2014) and was carried out in several steps: (1) In a first step, teachers’ central statements were sub-categorized. Central statements were identified by change in topics (e.g., a shift between teaching topics such as the use of digital tools to classroom management or a shift of the perspective of teacher to student needs). Thus, a sentence could be split up into several central statements as well as a statement could comprise several sentences and each statement could be assigned to one category. (2) The statements were then grouped into a category scheme developed in a previous study for the systematization of sources of teacher well-being (Hascher and Waber, in review) that addressed the various predictors of teacher well-being, e.g., individual dispositions, working conditions, teacher-student relationship, or school climate. Additional categories needed to be defined for statements related to specific influencing factors in the context of the COVID-19 pandemic such as the unpredictability of a pandemic. The coding scheme (see Table 1) covered two main areas, namely objective and subjective factors. Objective factors were defined by their independence of an individual interpretation and a neutral description of characteristics such as teacher age, year of teaching, school form, or school size; subjective factors were defined by individual perception of the self (e.g., self-efficacy, competence) and the work context (e.g., collegial support, teacher-student relationship). Objective and subjective factors were each subdivided into general individual, work-related individual, and work-related contextual sub-factors. General individual factors include factors related to a persons’ living condition (objective) or a person’s character and dispositions (subjective). Work-related individual factors include years of teaching or class composition (objective) and subjective factors such as motivation, self-efficacy, or competences (subjective). Work-related contextual factors include the resources and organization of a school (objective) and experienced work-related contextual characteristics of a school such as teacher cohesion, principal support, or student motivation and well-being (subjective). For example, if a teacher referred to her/his many years of professional experience, this was classified under the category “objective factors, individual work-related.” If a teacher mentioned that her/his work-situation had been impaired through a lack of support in the school faculty, this was categorized as “subjective factors, work-related contextual.” (3) In a next step, it was specified within the categories whether the example mentioned was perceived as supporting or maintaining (S) or deteriorating (D) teacher well-being. Few statements were coded as ambivalent (A) as teachers reported both supporting/maintaining and deteriorating effects, e.g., student well-being could be supportive for teacher well-being as well as a concern for teachers that impeded their well-being.

**TABLE 1:** Coding scheme and results.

**Category**	**Sub-categories**	***Frequency and short verbal examples (D) deteriorating or (S) supporting teacher well-being***
*Objective factors (N* = *68; N_D_* = *41; N_S_* = 25; *N_A_ = *2)*		
General individual *N* = 8 (12%)	Socio-economic status, age, living situation	*N*_D_ = 6; *N*_S_ = 2 (D) “My *home situation is not suitable for working from home* and I had to move so much stuff from the school” (S) “I just think by my *age*, … I have become a bit serene.”
Work-related individual *N* = 20 (29%)	Years of teaching, class teacher, employment, class form, additional tasks	*N*_D_ = 8; *N*_S_ = 11; *N_A_ = 1 (D) “I was never credited that I still have a family at home and *could not teach only my part time 50%*, zero consideration was given to this.” (S) “It was easier for me as I teach *first* graders. This is less stressful than fifth graders.”
Work-related contextual *N* = 40 (59%)	Student background, school resources, political strategies, work-holiday times, changes of the profession due to the pandemic	*N*_D_ = 27; *N*_S_ = 12; *N_A_ = 1 (D) “*I have a student who does not even have internet at home.”* (S) “It was helpful that school closure also included *regular spring holidays*.”
*Subjective factors (N* = *547; N_D_* = *199; N_S_* = 337; *N_A_ = *11)*		
General individual *N* = 118 (21%)	General health, personal characteristics (Big 5), coping strategies, character strength, emotion regulation, serenity, self-care	*N*_D_ = 26; *N*_S_ = 91; *N_A_ = 1 (D) “I had the feeling that *I cannot cope with all these challenges* due to the pandemic” (S) “P*ersonally* I am actually *doing well*.”
Work-related individual *N* = 240 (43%)	Word-loads, job demands, job satisfaction, professional attitudes, professional motivation and engagement, feelings of competence, self-efficacy beliefs, sense of commitment, professional challenges, consulting, autonomy, role as teacher, work-family balance, technical skills, new learning experiences	*N*_D_ = 128; *N*_S_ = 107; *N_A_ = 5 (D) “But the further the situation progressed and the more hopeless the situation became, that the child would connect and that I could reach it, *I felt like I was losing some*.” (S) “I *love my job and liked the challenges* of distance teaching.”
Work-related contextual *N* = 198 (36%)	Cooperation among teachers, peer conflicts, contact to faculty, relationship to students, principal support, relationship to parents, social appreciation, societal expectations, flow within school days, unfamiliar situations, structural resources	*N*_D_ = 45; *N*_S_ = 139; *N_A_ = 5 (D) “We had so *many problems in the team.*” (S) “I was just responsible for the subject of sport, someone else on the subject of French and *then we all did the dossiers and then everyone said the same thing, so you felt very carried in the faculty.”*

*N_*A*_ = Example could not be coded as deteriorating or supporting as both aspects were addressed (example for subjective work-related individual: “One day, I was exhausted and tired, the other morning I felt good and motivated … there was no consistency in my well-being and also the children’s well-being”; example for subjective work-related contextual: “Some children managed the situation well and others were lost”).

The data-set consisted of *N* = 615 teacher statements and was analyzed with MAXQDA. At the beginning of the coding process, two independent raters coded 30% of the statements with a good interrater-reliability of Kappa = 0.86. Discrepant results were discussed and according statements were added as examples to the coding list in order to guide the subsequent coding process. All remaining 70% of the statements were coded by one of the two raters.

## Results

In the following sections, main results and most relevant factors are presented and illustrated by teachers’ direct responses to the interview questions. Participant number (P01–21) and question number (Q1–5) are indicated with the examples, i.e., P21-Q3 indicates an exact quote from participant number 21 to question 3.

### Primary Teacher Perceived Well-Being During School Closure

In general, it can be said that the 21 teachers reported a high level of well-being. On a scale of 1 (very low level of well-being) to 10 (very high well-being) all teachers indicated their well-being level above 6, and almost half chose the value 8. However, teachers agreed that school closures had a severe impact on everyday school life and that such experiences could affect their well-being as can be seen in the following examples.

“At the beginning, when everything was so insecure, it was very difficult, you like hung in the air. And then suddenly there was no school, … until Sunday, we didn’t know what was going on, and that was kind of unsettling or unreal. At first I almost didn’t believe that something like this could happen” (P05-Q02).

“At the very beginning when we were informed about school closure, I was extremely surprised and felt lost …. Also, when we started with the organization of distance teaching, my well-being went down again due to the high number of organizational tasks that are usually not related to my work as a teacher” (P14-Q02).

“Yes, that was certainly at the start of this phase. Until I started the whole process with this distance learning, that the children could then really log into the different platforms. The whole electronic stuff and – also related to my well-being – to realize when parents are struggling with their children and I can’t do anything” (P12-Q02).

Apart from the general negative evaluation of the sudden school closure, it became evident that each teacher had experienced the situation differently during the school closure. Despite the broad agreement on the evaluation of school closure as challenging and the high demands that school closure has placed on teachers, a variety of difficulties and resources in dealing with these challenges were mentioned. In general, subjective aspects were particularly decisive for teacher well-being. Although objective factors, such as employment conditions or policy prescriptions, played a significant role, in almost 90% of the statements, teacher well-being was associated with subjective factors such as personal conditions, available or missing psychosocial resources or social support. In total, teachers reported fewer deteriorating factors (39%) and more supporting factors (60%). However, the results also show that teachers report more deteriorating objective factors (60%) than supportive objective factors (37%), while the opposite can be found for subjective factors (deteriorating 36%; supportive 62%).

### Objective Factors Deteriorating Primary Teacher Well-Being

The period of school closure has been an incisive experience with a number of negative facets that could have had a degenerative influence on teacher well-being. Teachers mentioned that, in the case of *objective individual factors (general and professional)*, the double burden of work and family care (*n* = 4), as well as part-time employment (*n* = 4), affected professional well-being. For example, one participant spoke about family responsibilities.

“That was perhaps the hardest thing. At home with the children … but at school I had to work a lot more than my assignment of less than 50%. And also, the expectation that I had to be available everyday for the school. My children also had appointments, zoom meetings, or they had to get material, they had to do their tasks” (P19-Q02).

Regarding *objective work-related contextual factors*, specific attention was given to diversity due to federalism (*n* = 11), challenging students (*n* = 5), insufficient school resources (*n* = 4), and changes of the teaching profession due to the pandemic (*n* = 4). The lack of a common strategy of schools and cantons and the navigation with the new professional technical tools could reduce teacher well-being as indicated in the example below.

“I think clear instructions, for whole schools, or entire cantons or Switzerland-wide would also have been very nice. Every school or canton does something different and every teacher does a little bit as it does for them. I find this very difficult and also very difficult for the parents, who then have different children in different classes” (P07-Q04).

### Subjective Factors Deteriorating Primary Teacher Well-Being

From the teacher’s point of view, dealing with the crisis or distance learning was closely linked to their *general individual factors.* Well-being was impaired when emotional stability and resilience were low (*n* = 11), the uncertainty with the switch to distance learning was high (*n* = 5), or if teachers reported pre-existing health issues (*n* = 4). Low emotional stability during the switch to distance teaching reads as follows:

“In various ways, not just professionally, just in general … the knowledge that I now have to switch to distance teaching has caused my anxiety… I felt like I couldn’t handle that” (P15-Q02).

Among the *subjective individual work-related factors*, increased professional challenges (*n* = 33), heavy psychological stress (*n* = 25), a lack of experience of competence and self-efficacy (*n* = 18) and high workload (*n* = 17) were reported as negative influences. The shift in responsibility for children’s learning from teachers to parents (*n* = 10) also affected teacher well-being. High workload was described as follows:

“During the 8 weeks when the schools were closed, that was a dip and wake up after 8 weeks. It was just heavy. It wasn’t all bad, however, it was just insanely intense, I was working from the morning at 7, when I got up, always until late in the evening” (P16-Q02).

In addition, specific characteristics of the teaching profession (*n* = 17) proved to be detrimental to well-being.

“I just had the feeling that it’s still the same with the fact that teachers are poorly supported and try to do everything alone. You talk about it a lot and do quality development and courses for fostering cooperation, but this has not yet sustainably changed our school culture” (P02-Q05).

Also, the general uncertainty and uncontrollability of the situation (*n* = 14) played an important role.

“This uncertainty was a burden for me, because this class will transit to another school in summer and I was totally unsure how to reach the learning goals with the students. I found that difficult” (P06-Q02).

Among the *subjective work-related contextual* factors, key topics of discussion included missing work structures in the school (*n* = 8) and lack of social and professional support of the school management (*n* = 5):

“We have not been optimally supported by the school management either. I can honestly say that we really didn’t know what was going on for a long time” (P11-Q02).

Negative experiences were amplified when social relationships, specifically the relationship with students (*n* = 8) and parents (*n* = 5) and the parent-child relationship were unsustainable for the specific situation of distance learning (*n* = 5) as expressed in the following examples.

“The students did not work at home and they didn’t come to school. I was there in the school and they didn’t come to get the help I provided. The parents didn’t support me, they said that the students just don’t listen to them anymore, at 12–13 years old. It was such a low point where I noticed, I can’t go any further” (P11-Q02).

### Objective Factors Supporting Primary Teacher Well-Being

A few objective factors turned out to be relevant for the support of teacher well-being, among them education grade (individual work-related, *n* = 11) and a high IT-standard at school (work-related contextual, *n* = 6). It was also positively noted that the school closure period included school spring holidays (work-related contextual, *n* = 3). As can be seen in the following example, teachers also felt relaxed when school closure did not interfere with student selection and allocation (*n* = 8).

“I teach a 6th grade. I know the students well, I know the parents well, and the selections for transitions into secondary school were already done, so I was able to go into this school closure time quite relaxed” (P09-Q02).

### Subjective Factors Supporting Primary Teacher Well-Being

Subjective *general individual factors* included competences and personal characteristics that are generally important for managing life challenges and being able to cope well with stress and multiple demands. Such competences and characteristics include character strengths (*n* = 12), emotional stability (*n* = 11), and serenity (*n* = 9). Resilience (*n* = 17) was demonstrated as will and confidence to succeed under adverse conditions as shown in the following example:

“I knew this situation was new to everyone. No matter what position you are in school and I knew it would be good, no matter what, there are solutions. I had few concerns or fear or whatever” (P01-Q02).

Self-care (*n* = 14) also played an important role in maintaining and promoting well-being. It was important that teachers paid attention to themselves and focused on activities that promoted their well-being.

“Yes, now I’m using this phase, that I’m just in the garden and in the house… I’ve been able to move forward and have just distracted myself a bit from this very unique situation” (P21-Q02).

Of equal importance were stress coping strategies (*n* = 10) and positive attitudes toward the teaching profession (*n* = 9). Successful coping reads as follows:

“First, I struggled and then I noticed, I can’t change it, it’s just like that and I just have to let it go and, yes, every kid has just his environment and it’s going to come good … the courage to accept the gaps somehow” (P21-Q03).

Among the *subjective individual work-related* factors that helped to maintain or promote well-being, positive experiences with new forms of teaching (*n* = 19, see example), feelings of competence and self-efficacy (*n* = 16) and job satisfaction (*n* = 11) were most frequently described.

“And then I started to create learning videos in every subject, and I really got into flow. I was really always at school, from 8 in the morning to 5 in the evening and just filmed and cut things together. In the beginning of course, it took forever until I was more experienced. But it was so much fun” (P10-Q02).

Being well organized with a clear work schedule (*n* = 17) also proved to promote teacher well-being.

“I think it also helped me that my everyday life continued as normal, because I went everyday to school. I have my own room here and here I met with my work colleagues and we did everything together. So, my everyday life went on pretty normal, just without the kids” (P03-Q02).

In addition, teachers capitalized on their high level of professional motivation and engagement (*n* = 10).

“It’s actually my way, I think I love being a teacher. Yes, sometimes it’s a lot, but somehow it keeps me alive, I feel” (P04-Q01).

Teacher well-being also was supported when teachers accepted the situation not as a hindrance but as a challenge (*n* = 7).

“You have to think about what is best to help the situation. I just discussed with my team teaching partner and the special needs teacher on what we can do for the children. We really just thought, who are the children who need the help most” (P08-Q02).

*Subjective work-related contextual factors* primarily concerned the social and relational aspects of teachers’ work. Positive relationships with students (*n* = 34) and parents (*n* = 21) were of high importance. Also, participants described the way students promoted teacher well-being through signs of personal relatedness (*n* = 15) and parental recognition and gratitude (*n* = 12) as shown in the following example:

“The relationship with the families, with the children themselves and also with the parents, has become much more intense. Every week we called the students at home (besides the phone times that we had anyway) and the families had my mobile number (normally they only know my home number) … and then I really got the kids, they wrote to me sometimes ‘good night Mrs. X, sleep well”’ (P06-Q03).

Similarly, collegial support (*n* = 22) and leadership support by principals (*n* = 17) were important as can be seen in the following two examples.

“I think it was also the collaboration with other teachers who also provided a lot of teaching materials and it was a matter of course that you could exchange things” (P18-Q03).

“Thank god, the school management gave clear instructions. There was also a lot of help from the school management who simply said, ‘ok you do this and that,’ this gave certainty” (P04-Q02).

### Advice for Other Teachers

Teachers were asked about advice for other teachers in dealing with distance learning in a pandemic. Answers to this question were expected to reveal key ideas as to of what contributes to teacher well-being under very difficult circumstances. Each teacher gave several recommendations in dealing with such a challenging situation. The recommendations and strategies could be categorized into four main areas: personal mastery of the situation (professional skills and mental health), maintenance of social contacts with children and parents, cooperation with colleagues, and good administration.

Almost all teachers (*n* = 18) gave advice on how to keep personal mastery of the situation: to pay attention to one’s own work-life balance, to improve technical and instructional skills, to exercise, to set realistic goals, to set structures, to practice calmness and distancing, and to maintain self-confidence as expressed in the following example.

“The digital preparation is really important. I think that in today’s time you have to use these media, you control them, the different channels of course and then at the class level, that this is well structured, that the flow of information is clear. Then the digitization of the teaching material is very important and the communication, quite clearly who does what and when. Then, of course, the personal condition, to organize yourself at home so that you can also do a bit of sports” (P17-Q04).

Advice also frequently referred to social contact with students and parents (*n* = 11) and how maintaining and even increasing contact was needed.

“If you can communicate with the kids just now via teams or zoom, or somehow, then I think that’s a very good thing, that you can talk to the kids that you see them, to share something with them. This depends on age, of course, but I really think that’s the ideal if you can just see each other and talk to each other. The other way, simply giving working sheets and things home, is not satisfying. The important thing is really to stay in touch with the students and parents” (P04-Q04).

Teachers also recommended collaboration with colleagues (*n* = 5) which made it easier not only to solve problems, but to minimize loneliness.

“It is essential to collaborate with colleagues. It is so important that you can ask someone for support and feedback, especially if you have little experience with the school material and then you can meet several times until you get a feeling for the needs of the children. Then, you are able to help the children, support them, accompany them. There is a risk that you will become lonely very quickly if you fail to connect yourself with students and parents and colleagues” (P20-Q04).

From a more technical perspective, participants highlighted the importance of having correct data and contacts for parents (*n* = 3). Obtaining and updating parents’ contact details early on had proved to be a necessity together with careful instructions for both students and parents regarding online study.

“In our school, a lot of teachers said that we don’t have the contact details of the parents at all. In my case, it was easy because I had the data. When the school, however, wanted to send emails, it went back to half. So, I’d say if you have a new class personal data, mobile-phone numbers, email, that’s a must” (P13-Q04).

## Discussion

This study aims at understanding Swiss primary school teachers’ well-being during school closure due to COVID-19 pandemic in spring 2020. It was expected that the sudden school closure and the unprepared move to online teaching would lead to stress and discomfort among teachers and as consequence would have a negative impact on teacher well-being. Accordingly, this research also aimed at understanding how teachers react to restore or maintain their well-being in the face of adversity and the implications that can be drawn from these strategies for schools in order to support teacher well-being.

Results confirm that teachers faced severe professional challenges. Distance learning was neither a part of the societal culture nor the school culture, and so the move from face-to-face teaching to online teaching turned out to be a critical professional life event for all primary teachers (as shown in other studies, e.g., Klapproth et al., 2020) causing medium to high stress levels. Although they felt challenged by distance teaching, primary teachers in this study generally reported medium to high levels of well-being, similar to findings on general life quality of a representative Swiss sample 2 weeks after lockdown during spring 2020 (Moser et al., 2020). This result seems surprising as, according to the theory of critical life events (Filipp and Klauer, 1991; Parker et al., 2012), it was expected that the aversive and challenging experiences caused by school closure and distance teaching would hamper teacher well-being. As one explanation, the hedonic adaptation process (hedonic treadmill; Eysenck, 1990) can be taken into account. Teachers’ well-being might have settled back to its pre-pandemic level as was observed with other important life events (Diener et al., 2006). Based on our data, however, this unexpected finding might be better explained by the dominance of supportive factors that teachers reported during the interviews. Specifically, subjective individual factors such as self-efficacy and motivation and subjective factors that were related to the work context such as collegial support and social relationships to students and their parents seemed to empower teachers in coping with the difficult situation. With regard to the Job-Demands-Resource Model (Demerouti et al., 2001; Bakker and Demerouti, 2017) teachers could experience opportunities for personal growth through social support as well as positive experiences with new forms of teaching that contributed to a motivation process. Although teachers also reported an array of negative factors that affected their well-being, they seldomly seemed to experience a strain process. In line with Tadić Vujčić et al. (2017) the findings suggest that teachers appraised the difficult and new situation rather as challenge demands than as hindrance demands. It has to be noted, however, that teachers’ positive evaluation may also be associated with a relief at the relatively short time period of school closure and also might fluctuate as was found in a diary study on university teacher well-being (Beltman et al., in press).

Despite the positive ratings of well-being, it should not be disregarded that primary teachers reported a plethora of factors that negatively affected their well-being. Interestingly, technical problems as reported in several other studies (Alea et al., 2020; Alves et al., 2020; Klapproth et al., 2020) did not turn out to be a major issue. Instead, work-load, social distancing and feelings of lack of competence and self-efficacy were among the most aversive aspects of distance teaching. Workload seemed to be more pronounced during the pandemic and a lack of confidence in teacher’s own capacities confirmed the negative influence on well-being found in general studies on teacher well-being (e.g., Aelterman et al., 2007; Vazi et al., 2013). This result needs specific attention as teachers in Switzerland have high weekly workloads compared to other European countries (for lower secondary teachers see European Commission/EACEA/Eurydice, 2021). Given the fact that primary teachers tend to work part time and to cooperatively teach a class, extra workload might be a specific burden during the pandemic as cooperative distance teaching may consume additional resources. The loss of social relationships that was specific for the pandemic situation impeded well-being thus supporting the critical role that social aspects play for teacher well-being in general (Wong and Zhang, 2014) as well as during distance teaching.

The analysis of data differentiating objective variables that describe a teacher’s working characteristics and situations in school, and subjective variables that cover a teacher’s individual characteristics and interpretation of the working situation in school, proved to be helpful. It became evident that a negative influence was more pronounced among objective variables. Issues such as double burden of family and school demands, high expected workload, inconsistent political strategies and students that needed special support in online learning, are examples that impeded teacher well-being. Within objective and subjective variables, work-related contextual aspects such as school resources, collegial support or leadership support confirmed their high valence to teacher well-being during the pandemic. These results contribute to research that highlighted the importance of schools’ organizational characteristics such school climate (Burns and Machin, 2013), organizational justice (Capone and Petrillo, 2016), or trust in principals (Berkovich, 2018) for teacher well-being. Also, they confirm the importance of teacher emotions for school effectiveness (Leithwood, 2007).

Distance teaching called for new actions and teaching competencies such as designing tasks that students could solve at home or maintaining contact with the class and individual students via digital media. This new “teaching profile” that was forced by distance teaching was described in an interview study with 15 Indonesian primary teachers (Putri et al., 2020). The profile indicated that: Teachers needed to adjust the curriculum, they had to figure out how to create exciting learning environments for online learning, they had to give online feedback to students and their parents to support student learning and they had to adapt their assessment strategies. Thus, it could be expected that also during a pandemic, schools that foster professional learning would contribute to teacher well-being (Tang et al., 2018).

Subjective variables were revealed to be predominately positive for teacher well-being. Individual characteristics such as resilience, serenity, emotional stability, self-care and professional attitudes supported teachers’ well-being. As consistently found, resilience acts as a protective factor and a nurturing source when teacher well-being is under pressure as it helps teacher adaptation to an adverse situation (e.g., Beltman et al., 2011; Pretsch et al., 2012; Gu and Day, 2013; Brouskeli et al., 2018; Mansfield, 2021). In line with the “Job Demands-Resource Model” (Demerouti et al., 2001; Bakker and Demerouti, 2017) resilience may help to appraise challenging situations as opportunities for personal growth and to initiate a motivation process instead of a strain process. However, resilience may not exclusively be defined as a personality factor or an individual competence but can also be defined as an organizational feature. Organizational resilience represents a social construct that frames teacher effectiveness and support teachers’ needs (Gu and Day, 2007). Both facets of resilience, individual and organizational, can support the maintenance of well-being (e.g., Richards et al., 2016). On the other hand, work-related contextual risk factors such as inconsistent policies, unsupportive administration or lack of school resources (e.g., Beltman et al., 2011) as reported by the teachers in this study can hamper resilience and teacher well-being.

Task focused coping was found to foster teacher well-being (Soykan et al., 2019). In the present study, factors that lead to teacher well-being can be identified as approach strategies as introduced by Carver and Scheier (1998) in their multidimensional model of coping. The teachers reported strategies such as acceptance of the situation, seeking emotional and instrumental support, practicing positive reframing as well as active coping and to improve their planning. Thus, in accordance with an international study on language teachers’ coping strategies during the pandemic (MacIntyre et al., 2020), approach-coping strategies were associated with teacher well-being.

Of specific interest is the result that new teaching experiences induced by distance teaching were empowering for the primary teachers. Teachers shared examples of innovative instructional designs such as producing learning videos, or giving individual feedback to recorded samples of student exercises in a foreign language which enhanced teacher self-efficacy and, in turn, teacher well-being. As regards schools as organizations, these positive experiences were grounded in the high autonomy that is given to the teachers in Swiss primary schools. Accordingly, good working structures and work organization, professional leadership and a supporting teacher learning climate (Shoshani and Eldor, 2016) helped to foster teacher well-being. It seemed important, that these new experiences were supported by colleagues and principals and valued by students and parents. These findings bear high practical relevance as they highlight how teacher professional development needs to be supported.

This study has several limitations. Although a characteristic of qualitative research and also current research on schools in the pandemic, one limitation is the non-representative character of the sample and the small sample size. Although the 21 participants are working in 15 different schools, the snowball sampling technique may have intensified the selectivity of the sample. However, detailed individual appraisals of the school closure could be identified. Another limitation is the timing of data collection. Due to the high demands on teachers during the pandemic and after the reopening of schools as well as school holidays, teacher statements might be impacted by memory bias. We could not compare teachers’ well-being during the pandemic with their well-being before school closure and, thus, could not control for difference between their wellbeing experiences and *post hoc* reports. As we focused the interview on primary teacher well-being during the pandemic, findings might be only valid for this specific societal situation and primary education and be limited in terms of their *general* relevance for understanding teacher well-being.

Apart from these shortcomings, the results of this study contribute to the body of research that aims at understanding the factors that impede or support teacher well-being in challenging situations (Education Support, 2020) and, thus, contribute to the broader empirical evidence on teachers’ professional lives as well as teaching and school effectiveness. More specifically, this study confirms the importance of schools providing an organizational frame and professional home for primary teacher well-being and effectiveness. Research on teacher well-being needs to better acknowledge that teacher work is nested within schools (Schaffer et al., 2007) and, thus, schools play a major role in supporting teacher professional well-being. This was evident for distance learning related to the COVID-19 pandemic. Individual support of primary teachers along with high quality school leadership that helps to structure and organize distance teaching seemed to be key for their well-being. The specific difficulties of primary education such as heterogeneous classrooms and educating young children from diverse backgrounds that are unfamiliar with forms of academic learning outside of school setting may have been an extra demand for the teachers. This points to the crucial role that a school communities play for teachers and support both the idea of well-trained school leaders – for example regarding health education and professional guidance – and cooperative school structures that respond to teacher needs such as the need for relatedness, for example through common rituals and shared working time for collaboration that can be continued during distance teaching.

During school closure when teachers had to develop new skills, various forms of support were needed. Practical implications for school management and teacher education emerging from this research are to create conditions for a support system where teachers individually and collectively receive support and feedback according to their needs in developing new teaching skills and new forms of communication with children and parents, colleagues and principals. Communities of learners in strong organizations with supportive leadership are needed to maximize teacher development, effectiveness and well-being.

## Data Availability Statement

The datasets presented in this article are not readily available because data set is restricted to the Swiss education system. Requests to access the datasets should be directed to the corresponding author.

## Ethics Statement

Ethical review and approval was not required for the study on human participants in accordance with the local legislation and institutional requirements. The patients/participants provided their written informed consent to participate in this study.

## Author Contributions

This research is the result of an ongoing collaboration between the authors. TH, SB, and CM developed this specific interview project. TH, SB, and CM contributed equally to the rationale of the project and the development of the interview guideline. TH realized the interview study and developed the coding scheme that was evaluated by SB and CM. TH analyzed the data and wrote a first draft of the manuscript. SB and CM both edited the manuscript and added specifically to Sections “Theoretical Introduction” and “Discussion.” All authors contributed to the article and approved the submitted version.

## Conflict of Interest

The authors declare that the research was conducted in the absence of any commercial or financial relationships that could be construed as a potential conflict of interest.
